# Clinical efficacy of the first two doses of anti‐SARS‐CoV‐2 mRNA vaccines in solid cancer patients

**DOI:** 10.1002/cam4.5968

**Published:** 2023-04-28

**Authors:** Maria Silvia Cona, Agostino Riva, Davide Dalu, Arianna Gabrieli, Cinzia Fasola, Giuseppe Lipari, Giacomo Pozza, Eliana Rulli, Francesca Galli, Lorenzo Ruggieri, Elsa Masedu, Gaia Parma, Davide Chizzoniti, Anna Gambaro, Sabrina Ferrario, Maria Antista, Matteo De Monte, Maciej S. Tarkowski, Nicla La Verde

**Affiliations:** ^1^ Department of Oncology Sacco Hospital, ASST Fatebenefratelli Sacco Milan Italy; ^2^ Department of Infectious Diseases, Sacco Hospital ASST Fatebenefratelli Sacco Milan Italy; ^3^ Luigi Sacco Department of Biomedical and Clinical Sciences DIBIC University of Milan Milan Italy; ^4^ Laboratory of Methodology for Clinical Research Istituto di Ricerche Farmacologiche Mario Negri IRCCS Milan Italy

## Abstract

**Introduction:**

Cancer patients are frail individuals, thus the prevention of SARS‐CoV‐2 infection is essential. To date, vaccination is the most effective tool to prevent COVID‐19. In a previous study, we evaluated the immunogenicity of two doses of mRNA‐based vaccines (BNT162b2 or mRNA‐1273) in solid cancer patients. We found that seroconversion rate in cancer patients without a previous exposure to SARS‐CoV‐2 was lower than in healthy controls (66.7% vs. 95%, *p* = 0.0020). The present study aimed to evaluate the clinical efficacy of the vaccination in the same population.

**Methods:**

This is a single‐institution, prospective observational study. Data were collected through a predefined questionnaire through phone call in the period between the second and third vaccine dose. The primary objective was to describe the clinical efficacy of the vaccination, defined as the percentage of vaccinated subjects who did not develop symptomatic COVID‐19 within 6 months after the second dose. The secondary objective was to describe the clinical features of patients who developed COVID‐19.

**Results:**

From January to June 2021, 195 cancer patients were enrolled. Considering that 7 (3.59%) patients tested positive for SARS‐CoV‐2 and 5 developed symptomatic disease, the clinical efficacy of the vaccination was 97.4%. COVID‐19 disease in most patients was mild and managed at home; only one hospitalization was recorded and no patient required hospitalization in the intensive care unit.

**Discussion:**

Our study suggests that increasing vaccination coverage, including booster doses, could improve the prevention of infection, hospitalization, serious illness, and death in the frail population of cancer patients.

## Graphical abstract

Prevention of SARS‐CoV‐2 infection is crucial for cancer patients given their frailty and vaccination is the most effective method to achieve this goal. The present study aimed to evaluate the clinical efficacy of the first two doses of anti‐SARS‐CoV‐2 vaccines in treated solid cancer patients. The clinical efficacy was 97.4%. COVID‐19 disease in most patients was mild and managed at home; only one hospitalization was recorded and no patient required hospitalization in the intensive care unit.

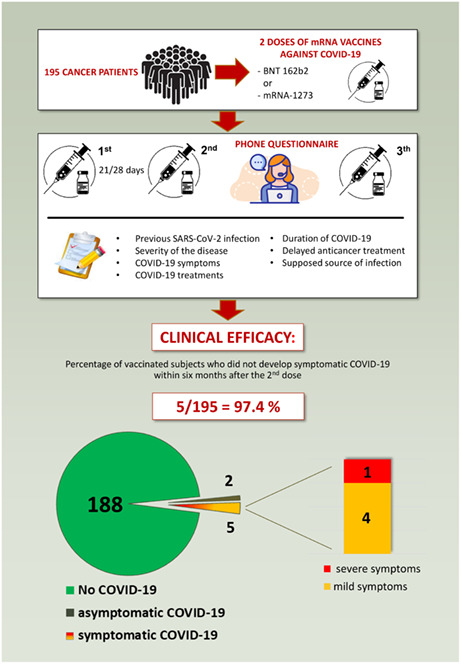

## 1 INTRODUCTION

On March 11, 2020, the World Health Organization (WHO), after assessing the severity and global spread of SARS‐CoV‐2 infection, declared the status of global pandemic.^1^, ^2^

Since then, there have been more than 600,000,000 cases of coronavirus disease‐2019 (COVID‐19) and 6,500,000 deaths worldwide, of which 178,000 occurred in Italy.^3^

To date, patients with cancer have showed an increase of SARS‐CoV‐2 infection rate (95% confidence interval [CI]: 8%–9%) with a twofold increased risk of adverse outcome (odds ratio [OR] for mortality 2.23, 95% CI: 1.82–2.94; intensive care unit [ICU] admission 2.39, 95% CI: 1.90–3.02) and severity of COVID‐19 (OR for hospitalization or severity of symptoms 2.08) compared to the general population.^4^, ^5^, ^6^, ^7^, ^8^

In the early days of the COVID‐19 pandemic, the National Health Systems imposed a suspension of nonurgent medical services worldwide. Many studies reported a downscaling of cancer treatment with an increased risk of impaired efficacy. In addition, the delay in cancer diagnosis and treatment could have jeopardized patient prognosis and long‐term population outcomes.^9^, ^10^, ^11^, ^12^

Many medical oncologists tried to protect patients from nosocomial contagion, through the reorganization of hospital spaces and the application of all prevention and mitigation procedures (triage at the entrance, the use of individual protection devices, social distancing measures, etc.). Anyway, the real “ace up the sleeve” in the fight against SARS‐CoV‐2 was the vaccine.^13^

In this dramatic scenario, the real change of course took place thanks to the launch of a global mass immunization strategy with anti‐SARS‐CoV‐2 vaccines. Since December 2020, Pfizer/BioNTech, AstraZeneca, and Moderna were among the first companies to develop anti‐COVID‐19 vaccines that were approved by the International Regulatory Agencies.

From January 2021, the most important international oncology societies advocated for the high priority of vaccination in cancer patients, in order to attenuate the harmful consequences of the pandemic. Several studies in very heterogeneous populations of cancer patients evaluated the immunogenicity of one or two doses of COVID‐19 vaccine.^14^, ^15^ In a previous study,^16^ we measured the antibody response to two doses of mRNA vaccines in solid cancer patients on active treatment. The seroconversion rate in patients with previous exposure to SARS‐CoV‐2 was comparable to that of healthy subjects (respectively 93.3% and 95%), but significantly lower in patients without previous infection (93.3% vs. 66.7%, *p* = 0.0020). Moreover, antibody response to vaccination negatively correlated with clinical variables of immune frailty, such as comorbidities, use of granulocyte‐colony stimulating factor (G‐CSF) and vaccine type. Besides, poor data are available in cancer population regarding the clinical efficacy of the vaccines, which is a parameter of protection from symptomatic disease.^17^

The aim of the present study was to evaluate the clinical efficacy after two doses of mRNA‐based anti‐SARS‐CoV‐2 vaccines in the population enrolled in the aforementioned study.^16^

## 2 MATERIALS AND METHODS

This is a single‐institution, prospective observational study conducted from January to December 2021 at Luigi Sacco Hospital in Milan.

We enrolled consecutive patients affected by solid malignancies, both in active treatment and in follow‐up, who received two doses of anti‐COVID‐19 vaccine (BNT162b2 [Comirnaty, BioNTech/Pfizer] or mRNA‐1273 [Spikevax, Moderna] vaccine).

In the period between the second and third dose, patients were contacted through a phone call and were investigated with a predefined questionnaire in order to collect information regarding the clinical efficacy of vaccination. Demographic information, preexisting medical conditions, signs and symptoms, and clinical outcomes of a possible SARS‐CoV‐2 infection were recorded.

The collected data were:

1. Previous infection, demonstrated by nasopharyngeal swab testing for SARS‐CoV‐2 through reverse transcription‐polymerase chain reaction (PCR) or antigenic testing;
2. COVID‐19 symptoms: fever ≥37.5°C, cough, rhinorrhea, sore throat, arthralgia/myalgia, gastrointestinal symptoms, and dysgeusia/dysosmia;
3. Severity of the disease: asymptomatic, symptomatic managed at home, hospitalization, and admission in ICU;
4. Therapies: non‐steroidal anti‐inflammatory drugs (NSAIDs)/paracetamol, antibiotics, hydroxychloroquine, low molecular weight heparin (LMWH), monoclonal antibodies, antiviral drugs, no treatment;
5. Duration of COVID‐19 disease was defined as days from positive to negative SARS‐CoV‐2 test; it was stratified in the following three groups: 1–10 days, 11–30 days, or >30 days;
6. Delayed anticancer treatment equal or greater than 7 days;
7. Supposed source of infection: family/friends, public spaces, or hospital.

The primary objective was to describe the clinical efficacy of vaccine, defined as the percentage of vaccinated subjects who did not develop symptomatic COVID‐19 within 6 months after the second dose. The secondary objective was to describe the clinical features of the patients who developed COVID‐19, in particular: possible sources of infection, symptoms, severity and duration of disease, treatment administered, delay in the administration of oncological therapy.

The study protocol was conducted according to the principles of the Declaration of Helsinki. All the participants signed written informed consent before any study procedure.

## 3 RESULTS

One‐hundred ninety‐five cancer patients who had received two doses of anti‐SARS‐CoV‐2 mRNA‐based vaccine were enrolled. The median age was 64.1 years (Q1–Q3: 53.8–72.0) and 70.8% of the patients were female. Breast was the most common tumor site (51.3%) and most of the patients had metastasis (67.2%). The BNT162b2 vaccine was administered in 71.8% of the subjects. Among 166 patients on active cancer treatment, the vaccine was injected after one or more cycles of therapy in 86.7% of the individuals. Traditional chemotherapy (33.3%) and targeted therapy alone (35.4%) represented the most used treatments. Forty‐four patients (22.6%) had more than one comorbid condition. Chronic steroid (duration of therapy ≥3 months) and G‐CSF use (at any dose and schedule) were reported in 45.1% and 7.7% of patients, respectively (Table 1).

**TABLE 1:** Demographic and clinical features of cancer patients (*n* = 195).

Tumor site	*n* (%)
Breast	100 (51.3)
Gastroenteric	30 (15.4)
Lung	24 (12.3)
Genitourinary	15 (7.7)
Gynecological	17 (8.7)
Head and neck	2 (1.0)
Other	7 (3.6)
Tumor stage^a^	*n* (%)
Limited	62 (31.8)
Advanced	131 (67.2)
Therapy	*n* (%)
No therapy	28 (13.0)
Chemotherapy	65 (30.2)
Target therapy	69 (32.1)
Chemotherapy + Target therapy	24 (11.2)
Hormo*n*e therapy in metastatic disease	9 (4.2)
Comorbidity	*n* (%)
No	86 (44.1)
=1	65 (33.3)
>1	44 (22.6)
Steroids^b^	*n* (%)
Yes	88 (45.1)
Granulocyte‐colony stimulating factor	*n* (%)
Yes	15 (7.7)

Abbreviation: G‐CSF, granulocyte‐colony stimulating factor.

^a^ Not applicable for two patients.

^b^ Duration of therapy ≥3 months.

During the study period, seven (3.59%) patients tested positive for SARS‐CoV‐2; five cases developed symptomatic disease. Based on these results, the clinical efficacy of two doses of mRNA‐based vaccines against COVID‐19 was 97.4% (5/195).

The main characteristics of these seven patients are described in Table 2. Of note, none of them had COVID‐19 previously. Notably, among the group affected by symptomatic COVID‐19, three patients had not reached seroconversion after two doses of vaccine; the two seroconverted patients who developed symptomatic disease contracted the infection at least 5 months after administration of the second dose of vaccine.

**TABLE 2:** Demographic and clinical features of the seven cancer patients infected by SARS‐CoV‐2 after two doses of mRNA‐based vaccines.

Patient	Symptomatic					Asymptomatic	
	1	2	3	4	5	6	7
Age (years)	60	53	68	78	70	66	57
Sex	M	F	F	M	M	F	F
Type of vaccine	mRNA‐1273	BNT162b2	BNT162b2	BNT162b2	mRNA‐1273	BNT162b2	mRNA‐1273
Tumor site	Kaposi Sarcoma	Breast	Breast	Lung	Genitourinary	Gastroenteric	Breast
Tumor stage	Advanced	Advanced	Advanced	Advanced	Limited	Limited	Limited
Vaccine administration before antiblastic therapy	Yes	Yes	Yes	No	Yes	No	No
Cancer treatment	CT	Target	HT	CT + Target	No CT/Target	CT	CT + Target
Steroid use	Yes	No	No	Yes	No	Yes	Yes
G‐CSF use	No	No	No	No	No	Yes	No
Comorbidity	1	0	1	> 1	1	1	0
Timing between second dose of vaccine and positive SARS‐CoV‐2 test (months)	5	3	7	6	4	7	9
Development of COVID‐19 symptoms	Yes	Yes	Yes	Yes	Yes	No	No
Seroconversion after two doses of vaccine	Yes	No	No	No	Yes	No	Yes

Abbreviations: CT, traditional chemotherapy; G‐CSF, granulocyte‐colony stimulating factor; HT, hormone therapy; Target, target therapy.

The duration of COVID‐19 was 10 days in six patients; only in one patient the symptoms lasted for 30 days, causing the delay of over 7 days for the administration of oncological treatment.

Only one symptomatic patient required hospitalization due to disease severity. No patient needed invasive ventilation or hospitalization in ICU.

The other patients had mild illness and were managed at home and treated with ancillary and supportive therapy (NSAIDs or acetaminophen). Conversely, the only hospitalized patient received oxygen therapy, broad‐spectrum antibiotics, dexamethasone, remdesivir, and tocilizumab.

The majority of infected patients estimated that the family members or friends were the possible source of infection (Table 3).

**TABLE 3:** Characteristics of the clinical course of SARS‐CoV‐2 infection in 7 cancer patients after two doses of mRNA‐based vaccine.

Symptoms	Symptomatic patients					Asymptomatic patients	
	1	2	3	4	5	6	7
Fever	Yes	No	Yes	Yes	No	No	No
Cough	Yes	Yes	No	Yes	Yes	No	No
Rhinorrhea	No	Yes	No	No	Yes	No	No
Sore throat	No	No	Yes	No	Yes	No	No
Arthralgia/myalgia	No	Yes	Yes	Yes	No	No	No
Gastroenteric symptoms	No	No	No	No	No	No	No
Dysgeusia/dysosmia	No	No	No	Yes	Yes	No	No
Duration (days)	11–30	1–10	1–10	1–10	1–10	1–10	1–10
Delayed cancer treatment ≥7 days	Yes	No	No	No	No	No	No
Therapy	NSAIDs/paracetamol	NSAIDs/paracetamol	NSAIDs/paracetamol	Oxygen Dexamethasone Antibiotics Remdesivir Tocilizumab	NSAIDs/paracetamol	NSAIDs/paracetamol	NSAIDs/paracetamol
Source of infection (referred)	Family/friends	Family/friends	Hospital	Family/friends	Public space	Family/friends	Family/Friends

Abbreviation: NSAIDs, non‐steroidal anti‐inflammatory drugs.

## 4 DISCUSSION

The present study describes the clinical efficacy of two doses of SARS‐CoV‐2 mRNA‐based vaccines in a population of cancer patients both on active treatment and in follow‐up. After 6 months of follow‐up, 97.4% of patients did not develop COVID‐19. Our results are similar to those of previous studies that described a vaccine efficacy ranging from 62% to 94.4% before the emergence of the Omicron variants. This wide range was probably due to the heterogeneity of the research methodology. First, separate estimates of the effects of different variants of concern (VOCs) were not provided. In addition, the prevalence of VOCs was not considered, the study populations were not stratified according to the type of cancer (previous vs. active, solid vs. hematological) or timing of treatment (previous vs. ongoing treatment).^18^, ^19^, ^20^, ^21^, ^22^, ^23^, ^24^

Prevention of the severe form of COVID‐19 (defined as hospitalization, ICU admission or severity of symptoms) is crucial for cancer patients and vaccination is an effective strategy for achieving this goal.^25^

In this study, almost all of the patients (4 of 5 individuals) who developed COVID‐19 after the second vaccine dose had mild symptoms, for an average duration of 10 days, managed at home with symptomatic treatment.

Published data suggest that cancer patients have a deeper waning of the antibody titers after the primary vaccination cycle compared to the general population. Approximately 6 months after the second dose, several cancer patients have undetectable anti‐spike antibodies.^26^

It is noteworthy that in our study more than half of the patients infected by SARS‐CoV‐2 (57.1%) had not develop an adequate seroconversion after two vaccine doses, and all the patients but one developed SARS‐CoV‐2 infection over 4 months after the second vaccine. Therefore, they had probably reduced levels of neutralizing antibodies, thereby rendering them more prone to the infection.^16^

On the other hand, patients who contracted the infection more than 6 months after the vaccination or who did not seroconvert after the second dose did not manifest any symptom.

This observation suggests that, despite lack of humoral response to vaccination, a vaccine‐induced T‐cell response might protect from severe disease in patients receiving chemotherapy or immune checkpoint inhibitors.^27^, ^28^

This study has limitations. First, the implementation of extensive preventive measures by the Italian National Government during the observation period may have had a substantial effect on the observed low infection rates. In addition, it can be hypothesized that cancer patients and their caregivers, cognizant of their precarious state and apprehensive of contagion, may paid particular attention to the implementation of precautionary measures.^29^ Furthermore, due to the emergency situation, not all patients have been tested by systematic nasopharyngeal swabs. An additional limitation of the study is the absence of data regarding the prevalence of the different SARS‐CoV‐2 variants that infected the patients who developed COVID‐19 after vaccination. Indeed, during the early stages of the COVID‐19 pandemic, the first two doses of anti‐SARS‐CoV‐2 mRNA vaccines were designed to target the original strain of the virus and its early variants. Besides, the epidemic periodical reports on SARS‐CoV‐2 variant dissemination in Italy and the data from the SCIRE collaborative study (which tracked SARS‐CoV‐2 variants in Italy) suggest that the Alfa variant (B.1.1.7 and B.1.1.7+E484K) was unequivocally preeminent from January to June 2021, while the Delta variant (B.1.617.2) became predominant from July to December 2021. Other minor circulating strains were the Beta variant (B.1.351) and the Gamma variant (P.1).^30^, ^31^, ^32^

Vaccine efficacy against these variants was confirmed by several studies even if protective immunity rapidly declines over time.^33^, ^34^

Our study suggests that increasing vaccination coverage and implementing booster doses, in order to prevent infection, hospitalizations, serious illness, and death, is essential in a frail population such as cancer patients. High‐risk subjects may also benefit from additional mitigation measures that could reduce the risk of exposure. This suggestion could be extended to the general population in the global fight against COVID‐19.

Additional studies are warranted in order to assess the clinical efficacy in cancer patients who received booster doses of the anti‐COVID‐19 vaccine.

## AUTHOR CONTRIBUTIONS

**Maria Silvia Cona:** Conceptualization (equal); data curation (equal); project administration (equal); writing – original draft (equal); writing – review and editing (equal). **Agostino Riva:** Conceptualization (equal); funding acquisition (equal); methodology (equal); project administration (equal); resources (equal); supervision (equal); writing – review and editing (equal). **Davide Dalu:** Conceptualization (equal); data curation (equal); writing – original draft (equal); writing – review and editing (equal). **Arianna Gabrieli:** Data curation (equal); writing – review and editing (equal). **Cinzia Fasola:** Data curation (equal); writing – review and editing (equal). **Giuseppe Lipari:** Writing – review and editing (equal). **Giacomo Pozza:** Writing – review and editing (equal). **Eliana Rulli:** Formal analysis (equal); software (equal); supervision (equal); validation (equal); visualization (equal). **Francesca Galli:** Formal analysis (equal); software (equal); visualization (equal). **Lorenzo Ruggieri:** Data curation (equal); writing – original draft (equal); writing – review and editing (equal). **Elsa Masedu:** Data curation (equal). **Gaia Parma:** Data curation (equal); writing – original draft (equal). **Davide Chizzoniti:** Data curation (equal). **Anna Gambaro:** Investigation (equal). **Sabrina Ferrario:** Investigation (equal). **Maria Antista:** Writing – review and editing (equal). **Matteo De Monte:** Data curation (equal); investigation (equal). **Maciej S. Tartowski:** Writing – review and editing (equal). **Nicla Maria La Verde:** Conceptualization (equal); methodology (equal); project administration (equal); supervision (equal); writing – review and editing (equal).

## CONFLICT OF INTEREST STATEMENT

N.L.V. reports grant from Eisai; speaker bureau from GSK; travel expenses for conference from Gentili, Celgene, and Pfizer; advisory role from Novartis and Celgene; advisory role, travel expenses for conference from Pfizer; advisory board from MSD, Roche, Novartis, Astrazeneca, and Daiichi Sanyo. D.D. reports receiving grants from Gentili, travel expenses from Roche, Gentili, and Eisai. M.S.C. has served on the advisory board from Daiichi Sanyo. There are no other personal or financial conflicts of interest to disclose.

## ETHICS STATEMENT

The study protocol was approved by the Istituto Spallanzani Ethical Committee and AIFA (number 312 of the experimental registry 2020/2021) and conducted according to the principles of the Declaration of Helsinki. All the participants signed written informed consent before any study procedure. All subject data were anonymized as required by the Italian Data Protection Code (Legislative Decree 196/2003) and the general authorizations issued by the Italian Data Protection Authority.

## Data Availability

The data generated in this study are available upon request from the corresponding author.
